# The feasibility of collecting the physiotherapy outcomes airway clearance, physical activity and fitness for the Australian Cystic Fibrosis Data Registry

**DOI:** 10.1186/s12890-022-02141-5

**Published:** 2022-09-10

**Authors:** Angela Potter, Ben Singh, Emily Scutter, Carol Maher

**Affiliations:** 1 Physiotherapy Department, Women’s and Children’s Hospital, SA Health, 72 King William Rd, North Adelaide, SA 5006 Australia; 2 Allied Health and Human Performance, University of South Australia, GPO Box 2471, Adelaide, SA 5001 Australia; 3 Alliance for Research in Exercise, Nutrition and Activity (ARENA), University of South Australia, GPO Box 2471, Adelaide, SA 5001 Australia

**Keywords:** Physiotherapy, Airway clearance, Physical activity, Fitness, Health service improvement

## Abstract

**Background:**

Physiotherapy-related data, such as airway clearance techniques (ACTS), physical activity and aerobic fitness are not consistently included in international cystic fibrosis (CF) data registries. This study aimed to pilot the collection of ACTS, physical activity and fitness in a hospital CF clinic, as a step towards informing future national implementation.

**Methods:**

This study was undertaken in a CF clinic within a major tertiary hospital. Patients and families were invited to participate. Participants completed self-report questionnaires on ACT use and those aged ≥ 10 years completed a physical activity questionnaire (Core Indicators and Measures of Youth Health Survey) and aerobic fitness test (the A-STEP test). Participants also completed a survey to explore the tolerance and acceptability of the fitness test, and the perceived accuracy of the self-reported data collection.

**Results:**

Forty patients agreed to participate in the study (mean age = 9.8, SD = 4.1 years old; 52.5% female). All patients and/or families that were approached agreed to participate and completion rate for the ACTs and physical activity surveys was 98% and 100% (respectively). Completion rate for the fitness test was 55%, due to time constraints. Most participants agreed (≥ 90%) they could accurately provide ACT and physical activity data, and the assessments were tolerable and acceptable.

**Conclusions:**

Patients with CF and their families are able to and can acceptably provide physiotherapy-related data, and collecting self-report ACTs and physical activity data is highly feasibly during routine CF clinic visits. However, aerobic fitness testing using the A-STEP test may be less feasible in clinic environments, due to time constraints.

**Supplementary Information:**

The online version contains supplementary material available at 10.1186/s12890-022-02141-5.

## Background

Across the world, patient data registries collect and record standardised patient-related information including diagnoses, care processes, and outcomes [1]. Patient data registries offer a valuable source of disease treatment and outcome data which can drive improved patient care [2]. Further benefits of data registries include informing disease care guidelines, and monitoring outcomes of interventions and the safety of medications [2]. Practically, there needs to be a balance between the reliability, validity and specificity of data elements and the feasibility and acceptability of data collection by all patient registry stakeholders [3]. Hundreds of patient data registries exist around the world [1], including disease and intervention-based registries. Most high-income countries administer disease-based patient registries for cystic fibrosis (CF), a life-limiting autosomal recessive disease that affects the function of exocrine glands.

Physiotherapy is a cornerstone of CF treatment [4]. Physiotherapy often focusses on airway clearance techniques (ACTs), involving manual techniques such as percussions, postural drainage and autogenic drainage, which assist with clearing respiratory secretions and improving lung ventilation [4]. Various devices may also be used, such as positive expiratory pressure (PEP) and oscillating PEP devices, high frequency chest wall oscillation and intrapulmonary percussive ventilation [5]. In recent years, there has also been increasing emphasis on the role of physical activity and exercise, as an adjunct to ACTs [6]. Exercise has been shown to improve mucociliary clearance (secondary to increased ease of sputum expectoration, improved ventilation and respiratory flow) [7] and aerobic capacity [8] among individuals with CF. Participation in physical activity is also associated with a reduced rate of decline in pulmonary function [9], and improved quality of life [10], while improved fitness is associated with higher survival [11]. Despite the importance of physiotherapy in the treatment of CF, physiotherapy-related data, such as ACTs, physical activity and fitness are not consistently included in international CF data registries [12]. To our knowledge, data on ACTs are collected on the UK, USA and Canadian CF data registries, fitness data is collected on the UK registry only, whilst physical activity and fitness are not currently collected. The collection of holistic physiotherapy-related data (i.e., ACTs, physical activity and aerobic fitness) annually would allow cross-sectional and longitudinal analysis of data and comparison with other data recorded on the Australian Cystic Fibrosis Data Registry (ACFDR) such as lung function and survival. Analysis of all outcomes would allow robust recommendations regarding physiotherapy interventions across the CF phenotype spectrum.

We recently engaged with key Australian CF stakeholders to examine the importance of collecting physiotherapy-related data on the ACFDR. The peak body, CF Australia, and the ACFDR Steering Committee endorsed the study. Lead CF physiotherapists from every major paediatric and adult CF centre in Australia participated in the Delphi study, reaching consensus that physiotherapy-related data should be included on the ACFDR [12].

Though a number of barriers to collecting such physiotherapy data were identified, such as increased workload for clinicians and concerns about variability/validity/reliability of outcome measures, these were offset by the perceived benefits, such as the ability to benchmark and compare CF centres, understanding the outcomes of different physiotherapy interventions and to review trends in CF physiotherapy practice. A consensus (> 80% agreement) was reached for collection of data on ACTs (84% agreement), physical activity (89%) and fitness (95%), provided they were measured using feasible data collection approaches, namely self-reported surveys to gather ACT and physical activity data, and a field-based test to measure fitness. The clinicians acknowledged that more rigorous data would be collected using wearable activity monitors (for physical activity) and laboratory-based tests (for fitness), however, participants noted that these measures would be unfeasible for use with all CF patients on an annual basis. Borderline consensus (79% agreement) was reached for the collection of frequency of ACTs.

Whilst the Delphi identified strong support from physiotherapists for the routine collection of these physiotherapy outcomes for the ACFDR, the actual burden and feasibility of collecting such data in a clinical context is unclear. In addition, it is not known whether patients and families will be motivated/willing to provide such data. Therefore, this study aimed to pilot the collection of ACTs, physical activity and fitness data in a hospital CF clinic setting. Specifically, it aimed to examine the feasibility and acceptability of data collection, from the clinicians’, patients’ and caregivers’ perspectives, with a view of informing future national implementation.

## Aims/objectives

To assess the feasibility and acceptability of collecting ACTs, physical activity and aerobic fitness data during a CF clinic appointment in an outpatient clinic setting. The objectives were to:

1. Describe the collection of ACTs, physical activity and aerobic fitness data in a standardised format, with a view to annual collection for the ACFDR.
2. Assess the acceptability of methods to assess ACTs, physical activity and aerobic fitness among patients, families and physiotherapists.
3. Evaluate patient uptake and data completeness for providing ACTs, physical activity levels and aerobic fitness data.
4. Describe clinicians’ perspectives on the feasibility of assessing and recording this data in an outpatient clinic setting.

## Methods

### Study design

A mixed-method approach was used, combining cross-sectional quantitative data and qualitative data. The study was undertaken in a major tertiary hospital which provides CF care to all paediatric patients in the state of South Australia. Ethical approval was obtained from the Women’s and Children’s Hospital Ethics Committee (North Adelaide, South Australia; HREC/20/WCHN/64) and the University of South Australia Human Research Ethics Committee (2020/HRE01722). Written informed consent was obtained from all participants’ parents/guardians prior to enrolment, and children provided assent to participate.

### Patients and data collection

The study involved two participant groups:

1. *Patients*: Patients were eligible to participate in the study if they: (i) had a clinical diagnosis of CF; (ii) attended the Women’s and Children’s Hospital outpatient clinic; and (iii) were aged 18 years or under.
2. Senior CF physiotherapy staff from the Women’s and Children’s Hospital.

### Selection of instruments

#### ACT self-report survey

To our knowledge, whilst the UK CF registry data includes ACT type, no other CF patient data registries around the world assess ACT frequency. Therefore, a purpose-designed survey was created, aiming to capture the ACTs used and the frequency of ACT sessions during the past 7 days. The ACT item asked participants to select all ACTs used in the last 7 days from a multiple-choice list, with the option to add additional techniques. The ACT list was based on all of the ACTs in common use in Australia, as reported by the specialist CF physiotherapists during the Delphi study [12]. The number of physiotherapy sessions completed in the past 7 days was collected using a multiple-choice item (0 to > 14 times per week).

#### Physical activity self-report survey

An extensive literature review was undertaken, and physical activity measurement experts were consulted to identify candidate physical activity surveys with the following characteristics (1) proven psychometric properties, (2) ease of interpretation (e.g. scored in terms of duration of physical activity vs an arbitrary physical activity score out of 100), (3) preferably, brief, and (4) preferably, with items that are relevant to both children and adults (to allow continuity of data across the childhood-adulthood transition, and to prevent the need for separate child and adult versions on the ACFDR)*.* The Core Indicators and Measures of Youth Health Survey [13] was identified as best meeting this brief. This survey comprises of seven items (a 6-point scale for each of the 7 previous days), asking participants to recall the time spent in physical activity each day over the past week. Categorical responses range from ‘none’ to ‘more than 2 h’. This tool allows the daily and weekly physical activity duration and compliance with physical activity guidelines to be easily calculated. The tool has moderate validity relative to accelerometry (r = 0.47) and moderate reliability over time (ICC range = 0.41, 0.53) [13], which is comparable to other leading self-report physical activity instruments.

#### Aerobic fitness test

An extensive literature review was undertaken, and a leading CF physiotherapist clinician researcher was consulted to identify candidate field exercise tests. It was determined that the test should be progressive (to avoid ceiling effects) and that step tests were preferable to corridor tests, for space, safety and infection control reasons. The Alfred Step Test (A-STEP) [14] was selected as it has been developed in a leading, research-active Australian CF hospital to address issues of floor and ceiling effects for the CF patient population. The A-STEP test is an incremental step test which requires the participant to step up and down on a 20 cm step in time with a standardised, pre-recorded schedule, in which the metronome increases in speed every minute, lasting a maximum of 16 min. The final A-STEP level achieved by the participant (level 1–16) was recorded. In line with A-STEP recommendations, participants’ oxygen saturation levels and heart rate were monitored throughout the test, blood pressure was monitored before and after the test, and the participants were asked to self-report shortness of breath and leg fatigue every minute throughout the test and during the 5-min recovery period.

### Context

Recruitment of patients followed a pragmatic approach, within the context of a weekly CF outpatient clinic. Patients attending the CF outpatient clinic were approached for participation between February and June 2021. Patients attending the clinic were approached and invited to participate, unless they were experiencing an acute health or medical condition, or if they were unavailable (e.g., due to multiple appointments and having no time to participate). The clinic runs from 8:00am-12:30pm once per week, with up to 30 patients attending each week. Due to the risk of cross-infection, each patient is allocated a consultation room and all patient services are brought to them, with staff wearing full personal protective equipment when entering the patient's room. During a typical visit, patients often have multiple appointments at other areas of the hospital (e.g., for X-ray or blood tests) and are seen by members of the multidisciplinary team, including doctors, nurses, physiotherapists, psychologists and lung function technicians.

Upon arriving at the clinic, potentially eligible patients and their family/caregivers were approached and invited to participate in the study. Consenting participants were invited to complete a self-reported questionnaire gathering basic demographic information (gender and date of birth) and ACT information (described in further detail below). Patients and/or parents completed paper-based surveys themselves (generally, those > 10 years old completed the surveys themselves and those ≤ 10 years were assisted by their parents). Guided by findings from the Delphi study [12], participants aged 10 years and over were then invited to complete a physical activity questionnaire and the fitness test (the A-STEP test, described below). Finally, all participants were invited to complete an acceptability survey (described below). The CF physiotherapist entered the ACT, physical activity and fitness data into a database replicating the layout of the ACFDR. The physiotherapist recorded the amount of time taken for data entry and rated the burden experienced in collecting the information as high, medium, or low.

### Assessments of outcomes

#### Feasibility

Feasibility was evaluated based on uptake rate (the percentage of patients with CF who agreed to participate, out of the total number of patients invited), and completion rate (the percentage of participants who successfully completed the ACT survey, physical activity survey and fitness test, out of the total number who consented to participate). In addition, the lead CF physiotherapist at the Women’s and Children’s Hospital, who undertook the data collection, took field notes to determine their opinions regarding feasibility of ongoing collection of data for entry onto the ACFDR.

#### Acceptability

Likert-scale items were used to explore patients’ perspectives on the proposed assessments, including the acceptability of the length of time taken to complete the aerobic fitness test, and tolerance to test (‘strongly disagree’ to ‘strongly agree’), and the perceived accuracy of the self-reported ACT and physical activity data collection items. Participants were asked (yes/no) whether the measures were acceptable to them, whether they would be willing to complete them again next year, and if they would recommend them to other patients with CF. Optional open-ended items were used to capture additional comments.

### Sample size

This pilot study was a feasibility and acceptability study. Sample sizes of 12–50 participants are recommended for assessing the feasibility [15, 16]. Therefore, at study outset, we considered a sample size of 30–40 participants to be sufficient to evaluate the feasibility in this cohort. The study was conducted at the Women’s and Children’s Hospital CF weekly outpatient clinic over a period of 3 months.

### Statistical methods

Participants’ demographic information (age and sex) and results from outcome measures (ACTs, physical activity and aerobic fitness) were reported descriptively. Categorical data were reported as numbers and percentages (n, %), and continuous data were reported as means (SDs) and ranges.

Feasibility outcomes were calculated as follows:

- *Uptake rate*: Calculated as the percentage of patients with CF who agreed to participate, out of the total number of patients invited.
- *Completion rate*: Calculated as the percentage of participants who successfully completed the ACT survey (all patients), physical activity survey (patients aged 10 and over only) and aerobic fitness test (patients aged 10 and over only), out of the total number who consented to participate.
- *Protocol acceptability*: Perceived accuracy of self-reported data, willingness to repeat annually, recommendation for other cystic fibrosis patients.

Likert-scale acceptability data were collapsed for analysis, with response categories 1 and 2 collapsed to create a category ‘agree’, responses 4 and 5 collapsed to create a category ‘disagree’. Acceptability information was also collected as qualitative data, in notes recorded by the lead physiotherapist throughout the data collection. The open-ended responses were compiled and analysed thematically in a table [17]. The qualitative data was coded into categories related to the lead physiotherapists’ experience of the data collection procedure. In each category, codes were combined to create themes that reflected experiences related to collecting and recording the ACT, physical activity and aerobic fitness data. The analysis was conducted by two researchers (AP, ES).

## Results

### Participant characteristics

A total of 40 patients agreed to participate in the study. The mean age of participants was 9.9 (SD = 4.1; range = 2–17) years old, and 21 (52.5%) were female (Table 1). There were equal proportions of patients aged under 10 years old (n = 20, 50%) and 10–18 years old (n = 20, 50%). Mean physical activity for participants over the age of 10 years old was 373.6 (SD = 216.5) minutes per week (approximately 53 min per day). Mean level achieved on the A-STEP test for aerobic fitness was 10 (SD = 2.35) and ranged between level 7–14.

**Table 1:** Participant characteristics

	Male Mean ± SD (range), or n (%)	Female Mean ± SD (range), or n (%)	Total Mean ± SD (range), or n (%)
Age	9.4 ± 4.1 (2.0, 16.0)	10.4 ± 4.3 (2.8, 17.4)	9.9 ± 4.2 (2.0–17.4)
< 10 years	8 (41.1%)	12 (57.1%)	20 (50%)
10–18 years	11 (57.9%	9 (42.9%)	20 (50%)
Physical activity, mins/week	294.5 ± 262.9 (49–840)	440.8 ± 185.1 (180–810)	373.6 ± 216.5 (49–840)
Fitness, A-STEP Level^a^	10.6 ± 2.4 (8–14)	9.7 ± 2.7 (7–14)	10.1 ± 2.35 (7–14)

^a^Represents the level achieved on the test, on a scale of 1–16

### Feasibility

The participant uptake rate was 100%; all patients and/or families that were approached agreed to participate. Completion rate for the ACTs and physical activity surveys was 98% (n = 39 of 40) and 100% (n = 20 of 20) respectively. All participants (n = 20 of 20) consented to completing the aerobic fitness test. However, the aerobic fitness test was undertaken by 55% (n = 11 of 20). Nine participants did not undertake the test due to time constraints which prevented all patients from undertaking the test within their appointed clinic time.

Clinician's perspectives on feasibility of assessing and recording data was assessed using field notes taken by the lead physiotherapist. A summary of the field notes taken by the lead physiotherapist during the study is shown in Additional file 1: Table S1. When recruiting patients for the study, generally people were receptive and happy to participate, and many expressed surprise that such data were not already being collected on the registry. The notes indicated several instances where patients were not approached to participate due to them experiencing emotional distress at the time (e.g., if they were experiencing health or medical conditions). The ACT and physical activity surveys each took less than 5 min to complete, whereas the A-STEP test took 30–40 min to complete. The physiotherapist notes indicated that it wasn’t possible to complete the A-STEP with many patients, due to time constraints of the multi-disciplinary outpatient clinic setting. The time taken to enter each patient’s data into a database was approximately 1.5 min per patient.

### Acceptability

The acceptability questionnaire was completed by all participants (Table 2). Most agreed that they were confident they could accurately provide ACT data (97.5%, n = 39 of 40), confident they could accurately answer the number of ACT sessions (97.5%, n = 39 of 40), and that the time taken to complete the ACT survey was acceptable (95%, n = 38 of 40). With respect to the physical activity survey, most participants agreed or strongly agreed they were confident they could accurately complete the physical activity data (90%, n = 18 of 20) and that the time taken to complete the physical activity survey was acceptable (95%, n = 19 of 20). In relation to the aerobic fitness test, most participants who undertook the test agreed that they were able to tolerate the step test (90.1%, n = 10 of 11), and that the time taken to complete the step test was acceptable (81.8%, n = 9 of 11).

**Table 2:** Responses to acceptability survey

Outcome, n (%)	Strongly disagree	Disagree	Neither agree nor disagree	Agree	Strongly agree	Did not answer
*Airway Clearance (n = 40)*						
Confident to accurately complete airway clearance data	0	0	1 (2.5)	10 (25)	29 (72.5)	0
Confident to accurately answer the number of airway clearance sessions	0	0	1 (2.5)	11 (27.5)	28 (70)	0
Time taken to complete airway clearance survey was acceptable	0	0	1 (2.5)	13 (32.5)	25 (62.5)	1 (2.5)
*Physical Activity (n = 20)*						
Confident to accurately complete physical activity data	0	1 (5)	2 (10)	7 (35)	10 (50)	0
Time taken to complete physical activity survey was acceptable	0	0	1 (5)	6 (30)	12 (60)	1 (5)
*Fitness Test (n = 11)*						
Able to tolerate the step test	0	0	0	4 (36.4)	6 (54.5)	1 (9.1)
Time taken to complete the step test was acceptable	0	0	0	4 (36.4)	5 (45.5)	2 (18.2)
Overall testing (n = 37)	Yes	No	Missing			
Do you feel the current assessment is acceptable?	37 (100%)	0	0			
Will you be willing to do the assessment again next year?	37 (100%)	0	0			
Would you recommend the assessment to others?	36 (97%)	0	1 (3%)			

## Results of ACT survey, physical activity survey and aerobic fitness testing

Results of the ACT survey are shown in Table 3. The three most common ACTs used by patients were “PEP and Hypertonic saline” (n = 22, 55%), “exercise specifically for sputum clearance” (n = 14, 35%) and “pats and/or vibration and postural drainage” (n = 13, 32.5%). Twenty percent of patients (n = 8, 20%) reported using other techniques, which included trampoline, clarinet, bubble PEP, swimming bike riding, while one participant reported their lung function has been so good there was no need for airway clearance. Participants reported completing airway clearance an average of 7 (SD = 4.2) times per week (range = 0–14 times per week).

**Table 3:** Airway clearance, physical activity and fitness data collection

	Male (n = 19)	Female (n = 21)	Total (n = 40)
*Airway clearance and physiotherapy muco-actives, n (%)*			
None	3 (15.8)	3 (14.3)	6 (15)
PEP and Hypertonic Saline	11 (57.9)	11 (52.4)	22 (55)
PEP (no hypertonic saline)	1 (5.3)	4 (19.0)	5 (12.5)
“pats” (percussion) and/or vibrations and postural drainage	8 (42.1)	5 (23.8)	13 (32.5)
“pats” (percussions), no postural drainage	2 (10.5)	4 (19.0)	6 (15)
Hypertonic saline nebuliser only^a^	2 (10.5)	5 (23.8)	7 (17.5)
Exercise, specifically to help with sputum clearance	7 (36.8)	7 (33.3)	14 (35)
“active cycle of breathing”	4 (21.1)	3 (14.3)	7 (17.5)
Other	1 (5.3)	7 (33.3)	8 (20)
*Frequency of airway clearance, per week, mean (SD) (range)*	6.2 (4.7) (0–14)	7.1 (3.8) (0–14)	6.6 (4.2) (0–14)

*PEP* Positive expiratory pressure

^a^May/may not have included force expiratory techniques and directed coughing during nebulisation

## Discussion

### Summary of findings

The purpose of this pilot study was to evaluate the feasibility and acceptability of collecting physiotherapy-related data in an outpatient clinic setting, in the context of potentially gathering this data annually for all CF patients for inclusion on the ACFDR. Key findings indicated strong willingness from patients with CF and their families to provide this information, and that collecting self-report ACTs and physical activity data was highly feasible for collection during routine CF clinic visits. However, testing of aerobic fitness using the A-STEP test appears less feasible in a busy clinic environment, due primarily to clinician time constraints.

### Feasibility and acceptability

The high participation rate observed in our study, and strong willingness to provide physiotherapy-related data for the ACFDR on an annual basis, is likely to reflect that patients and caregivers view the collection of data on ACTs, physical activity and fitness as relevant, and important to the treatment of CF. Previous research has reported that families of children with CF consider physiotherapy treatment as an important form of treatment that should be prioritised [20]. Physiotherapists play an ongoing role in educating families and patients about CF and treatment techniques and promoting adherence to treatment [20, 21], and physiotherapy adherence is associated with better lung function, reduced hospitalisation, and improved quality of life [18, 19].

Excellent rates of data completeness were achieved for the ACT and physical activity surveys (98% completion). Further, most participants (95%) agreed that the time taken to complete each of these surveys was acceptable, and that they felt confident that they could accurately complete the survey questions about which ACTs they use, how often they perform ACT (physiotherapist) sessions, and how much physical activity they do. In contrast, the aerobic fitness test was only undertaken in just over half (55%) of the eligible patients (i.e. > 10 years of age), due to clinician time constraints. Though the A-STEP protocol goes for a maximum of 16 min, feasibility results suggested that it actually took around 30–40 min in total to administer, factoring in time for setting up the test, explanation, baseline measures, undertaking the test, and post-test monitoring. Most (82%) patients who completed the test agreed that the time to complete the test was acceptable. The physiotherapist described feeling strong time pressures due to the multi-disciplinary clinic setting, and not wanting to “hold up” other clinic staff. Thus, results suggest that the fitness test was acceptable from the patients’ perspective, but not feasible from the clinician’s perspective, in a multi-disciplinary outpatient clinic setting. It is possible that the test may be completed more successfully in a separate, subsequent clinic visit, however, this would require additional physiotherapy time resources.

### Strengths and limitations

A strength of this study was that it was driven by clinicians, and designed to answer a clinical question which has the potential to eventually improve the care and outcomes for CF patients across Australia. The study achieved a very high participation rate (< 5% of clinic patients were not approached due to acute medical reasons, including acute emotional distress, or not having time to participate, and all patients that were approached agreed to participate), improving confidence in its findings. Furthermore, the pragmatic nature of the study identified several barriers and other practical considerations for data collection in a real-world clinic setting, which ensures the findings are clinically relevant. An additional strength was that the peak body for CF in Australia and the ACFDR Steering Committee endorsed the study. Support from these key stakeholders will be an important for implementing data collection for the ACFDR in future.

Several study limitations should be acknowledged. Firstly, the study was conducted within a single CF centre. Initially, it was planned that the pilot study would be undertaken at the Adelaide Women’s and Children's Hospital (i.e. the current study), and then be replicated in several other Australian CF centres. Numerous participants in the Delphi study [12] had offered to contribute to this. At completion of this feasibility study, a meeting was arranged with the lead CF physiotherapists from across Australian CF centres for feedback of the results of this pilot and commence planning for the replication studies. However, when the results were presented, the other CF physiotherapists stated that their clinical experience indicated that findings would be similar (i.e., that collecting ACT and PA data using short self-reported surveys would be highly feasible in their clinical settings, whilst undertaking fitness tests which took 30–40 min per participant would not). Therefore, replication was deemed an unnecessary use of time and resources. A further limitation was that a small proportion (around 5%) of patients were not approached to participate in the feasibility study, based on the physiotherapist’s clinical judgement that it was not appropriate to add additional burden to that patient/family on that particular day, which may be consider a source of selection bias. The surveys were handed out to CF patients by a physiotherapist who was known to them, which may potentially introduce social desirability bias. Finally, certain aspects of feasibility (e.g., impact on multi-disciplinary team members, and length of time to administer the data collection tools) were gathered through the physiotherapist’s field notes. Including multi-disciplinary team members as an additional study participant group, and using a stopwatch to record data collection time, would have captured these data with greater rigour.

### Future directions

Based on the study’s findings, further discussions with other stakeholders to progress the addition of ACT and PA measures to the ACFDR are now underway. Though the self-reported items themselves are simple, adding ACT and physical activity outcomes to the ACFDR will involve considerable coordination and planning, including seeking approvals from the data custodians, the various CF centre directors and relevant ethics committees. The most appropriate route for data collection will need consideration, for example, whether completion of the surveys should be overseen/facilitated by physiotherapists in each centre (either paper-based on online), versus administering the surveys centrally using online surveys sent by SMS or email. Each of these approaches will have advantages and disadvantages, with in-person administration likely to achieve higher rates of data completeness, versus central online dissemination being lower in clinical burden, and allowing for additional patient reported outcome measures to potentially be collected.

This study, and the Delphi study preceding it, highlighted the challenge of balancing best-practice and measurement tools with practical considerations in clinical settings. For example, in the UK, annual exercise testing of CF patients is recommended, yet in Australia, it is currently far from routine practice. Regular exercise testing offers benefits such as enhanced patient monitoring, and at a group level, may inform treatment guidelines. However, this study (and the Delphi) highlighted the considerable clinical resources required for exercise testing, and it was deemed that it would be unfeasible to conduct annual testing with all CF patients within current clinician resources. In a similar vein, objective monitoring of physical activity has superior validity to self-reported physical activity tools [22, 23]. However, objective monitoring, again, entails considerable additional resources, in terms of equipment, and clinician time to administer the devices and interpret the data [24]. Given that adding physiotherapy outcomes to the ACFDR is a national-scale initiative, it seems that issues of feasibility and practical should be prioritised. However, in future, there is the potential to switch to objective PA measurement and add exercise testing, as these approaches become a more routine part of CF clinical practice in Australia.

### Conclusion

Physiotherapy-related data, such as data on ACTs, physical activity and fitness are not routinely included in international CF data registries, despite the importance of physiotherapy in the treatment of CF. Our findings indicated that patients with CF and their families have a strong willingness to provide this information, and that collecting self-report ACTs and physical activity data is highly feasibly during routine CF clinic visits. However, aerobic fitness testing using the A-STEP test may be less feasible in clinic environments, due to clinician time constraints. Further discussions with stakeholders to progress the inclusion of physiotherapy measures to patient data registries (such as the ACFDR) are warranted.

## Supplementary Information

**Additional file 1**. Summary of field notes taken from the physiotherapist during data collection. The physiotherapist took notes related to patient recruitment, staff availability, time available, willingness to participate, completing surveys, completing fitness test, and time taken to enter patient data. ACT: airway clearance techniques; PA: physical activity.

## Data Availability

The datasets used and/or analysed during the current study are available from the corresponding author on reasonable request.
