# Supplementary material for: The feasibility of collecting the physiotherapy outcomes airway clearance, physical activity and fitness for the Australian Cystic Fibrosis Data Registry
Source: BMC Pulm Med. 2022 Sep 10;22:342. doi: 10.1186/s12890-022-02141-5 (PMC9463726; doi:10.1186/s12890-022-02141-5)
Supplement: Supplementary file 1 — Additional file 1. Summary of field notes taken from the physiotherapist during data collection. The physiotherapist took notes related to patient recruitment, staff availability, time available, willingness to participate, completing surveys, completing fitness test, and time taken to enter patient data. ACT: airway clearance techniques; PA: physical activity. [file 12890_2022_2141_MOESM1_ESM.docx]

| Additional file 1: Table S1: Summary of field notes from lead Physiotherapist | |
| --- | --- |
| Name of Item | Notes |
| Patient Recruitment | Everyone approached was happy to participate.  Best to approach families at the start of their appointment or whilst waiting for lung function testing.  Occasionally people were not approached because they were busy with other clinicians.  In rare instances, people were not approached because of emotional distress (for example, for social reasons or clinical deterioration requiring admission). |
| Staff Availability | Physiotherapy staff were available, however often busy with other commitments. |
| Time Available | Parent/patient takes less than 5 mins to complete the ACT, PA and acceptability surveys  The step test takes 30-40 mins to complete, was often not conducted due to time restrictions in the clinic  Some patients declined to participant, due to time constraints. |
| Willingness to participate | Most patients approached were happy to participate, and all were willing to complete the testing again annually. |
| Completing ACT survey | Survey takes less than 5 mins to complete- appears to be very feasible |
| Completing PA survey | Survey takes less than 5 mins to complete- appears to be very feasible |
| Completing fitness test | Only 1-2 tests were completed per clinic, often consent was given but the test was not conducted due to time restrictions.  Testing took 30-40 minutes to complete- including equipment setup, explaining the progress, undertaking the test, cool down, pack up and cleaning.  Always under pressure to complete the test, often holding up other staff and often interruptions during the test by other staff.  Seems unlikely enough step tests will be done per clinic to go through all patients (10 years and older) annually.  Potential for equipment failure and patients wearing inappropriate footwear or clothing. |
| Time taken to enter patient data | Approximately 1.5 minutes per patient. |
